# Cenobamate, a New Promising Antiseizure Medication: Experimental and Clinical Aspects

**DOI:** 10.3390/ijms252313014

**Published:** 2024-12-03

**Authors:** Barbara Błaszczyk, Stanisław J. Czuczwar, Barbara Miziak

**Affiliations:** 1 Faculty of Medical Sciences, Lipinski University, 25-734 Kielce, Poland; barbarablaszczyk@op.pl; 2 Department of Pathophysiology, Medical University of Lublin, 20-090 Lublin, Poland; stanislaw.czuczwar@umlub.pl

**Keywords:** cenobamate, anticonvulsant effects, antiseizure efficacy, epilepsy, antiseizure medications

## Abstract

About 40–50% of patients with drug-resistant epilepsy do not properly respond to pharmacological therapy with antiseizure medications (ASMs). Recently approved by the US Food and Drug Administration and European Medicines Agency as an add-on drug for focal seizures, cenobamate is an ASM sharing two basic mechanisms of action and exhibiting a promising profile of clinical efficacy. The drug preferably inhibits persistent sodium current and activates GABA-mediated events via extrasynaptic, non-benzodiazepine receptors. Thus, its antiseizure potential is dependent on both reducing excitation and enhancing inhibition in the central nervous system. In experimental seizure models, cenobamate exhibited a clear-cut activity in many of them with promising protective indexes, with only bicuculline-induced seizures being unaffected. Randomized clinical trials indicate that combinations of cenobamate, with already prescribed ASMs, resulted in significant percentages of seizure-free patients and patients with a significant reduction in seizure frequency, compared to other ASMs in the form of an add-on therapy. Its greater antiseizure efficacy was accompanied by adverse events comparable to other ASMs. Cenobamate has also been shown to possess neuroprotective activity, which may be of importance in affecting the process of epileptogenesis and, thus, modifying the course of epilepsy.

## 1. Introduction

Apart from a variety of non-pharmacological therapies for epilepsy, antiseizure medications (ASMs) remain the main therapeutic method of treatment of this neurological disease [1]. The therapy of epilepsy is aimed at a complete reduction in seizures without accompanying adverse effects of ASMs, which can be hardly observed in a population of patients with epilepsy. Nevertheless, minimal expectations assume at least a 50% reduction in seizure frequency with tolerable adverse effects resulting from ASMs so that patients with epilepsy can lead a normal lifestyle [1]. About 30 or even 40% of patients with epilepsy may not properly react to a correctly tailored pharmacological therapy, according to the definition of drug-resistant epilepsy [2,3]. Drug-resistant epilepsy may be defined as failure of two properly chosen ASM schedules (in mono- or polytherapy) to achieve a seizure-free state [2].

ASMs exert their anticonvulsant effects via a couple of mechanisms leading to either an enhancement of inhibitory effects or reduction in excitatory events in the central nervous system [4]. Inhibitory effects may be expected in case of ASMs affecting voltage-operated ion channels or enhancing GABA-mediated synaptic events, whilst reduced excitation in the brain may result from blockade of ionotropic glutamate receptors or reduced synaptic release of excitatory neurotransmitters [5].

Chemically, cenobamate is a representative of alkyl carbamates, and in 2019, it was approved by the US Food and Drug Administration and by the European Medicines Agency (EMA) in 2020 for clinical use in adult patients with focal seizures [6,7,8].

With respect to cenobamate’s efficacy in animal models of seizures in mice, this ASM is active against maximal electroshock-induced convulsions, 6 Hz (44 mA)-, pentylenetetrazol- and picrotoxin-induced seizures, but inactive against bicuculline-induced convulsions. Among other ASMs, felbamate (structurally related to cenobamate) and valproate exhibited similar anticonvulsant efficacy, with the remaining ASMs exhibiting less pronounced efficacy [6].

The purpose of this review was to compile and update the state of knowledge on one of the newest ASMs, cenobamate. Drawing on a number of both original and review publications, this article presents the drug’s unique mechanism of action and its pharmacokinetics and pharmacodynamics. In addition, the present review collected results from experiments with cenobamate in both preclinical (in various animal models of seizures and behavior) and clinical studies (including a meta-analysis), which are also presented in the form of clear tables.

## 2. Search Strategy and Selection Criteria

The article search was carried out in PubMed, Google Scholar and Web of Science databases and the keywords were as follows: antiepileptic drugs (or ASMs), cenobamate and mechanisms of action (pharmacokinetics, drug interactions, efficacy in animal models, behavioral tests, and clinical data), drug load, and overtreatment of epilepsy. The search was preferentially confined to articles published in the time frame of 2018–2024.

## 3. Mechanism of Action

Studies have shown that the drug is distinguished by a multifaceted mechanism of action. Nakamura et al. [9] evaluated the “effect of cenobamate on voltage-gated Na+ channels in acutely isolated rat hippocampal CA3 neurons using a whole-cell patch-clamp technique”. This research group showed that this ASM did not significantly alter the peak amplitude, onset time, or decay time constant of transient sodium current (INaT)_,_ suggesting that the drug has little effect on “the peak component of transient INaT induced by brief depolarizing step pulses”. On the other hand, it was shown that cenobamate simultaneously exhibited a strong inhibitory effect on the persistent component of INa (INaP) and on voltage-ramp-induced current in a concentration-dependent manner, with an IC_50_ value of 53.1 μM. Also compared with carbamazepine and lamotrigine, cenobamate had greater preference for INaP inhibition than these ASMs and other drugs already known, which may explain the differences in efficacy and tolerability between them. Moreover, cenobamate significantly shifted the steady-state fast inactivation relationship toward a hyperpolarizing range, indicating that the drug binds to voltage-gated sodium channels at the inactivated state with a higher affinity. It was also observed that the drug accelerated the development of inactivation and delayed return after inactivation of the voltage-gated sodium channel. Further experiments with a current clamp showed that cenobamate hyperpolarized membrane potentials in a concentration-dependent manner, increased the threshold for generating action potentials and decreased the number of action potentials evoked by a depolarizing current injection.

The above experimental results can explain the anticonvulsant activity of cenobamate, and the analysis of the drug doses used allows us to conclude that the above effect can be obtained under therapeutic conditions using a dose range of 100–400 mg/day [9].

In conclusion, confirmed action on voltage-gated sodium channels, through a pronounced action on persistent currents (INaP) rather than INaT, increases inactive channel states (unlike some ASMs, including lamotrigine, felbamate or carbamazepine, which act on transient sodium current). By blocking the sustained sodium current and enhancing the inactivated state, cenobamate normalizes the seizure threshold and prevents neuronal hyperexcitability. In addition, it acts as a positive allosteric modulator at synaptic and extrasynaptic GABAA receptors regardless of the binding site of benzodiazepines. As a result, it modulates phasic (Iphasic or inhibitory postsynaptic current) and much potently tonic (Itonic) currents, which results in the production of inhibitory events in the brain [10,11,12]. In comparison, benzodiazepines act selectively on α subunits (α1–α3, α5), β subunits (β2, β3) and the γ2 subunit, which affects Iphasic inhibition [13,14], while barbiturates act on δ subunits, which enhances Itonic inhibition [15,16]. In summary, mechanisms of action of this ASM discovered so far encompass a distinct blockade of persistent sodium channels and potent augmentation of extrasynaptic tonic GABA-ergic currents, their effect on synaptic GABA-ergic current being much weaker [6,7,9]. There is a striking difference between cenobamate and other ASMs (carbamazepine, lamotrigine, felbamate, phenytoin, topiramate, or valproate) in that other drugs preferably block transient sodium current (carbamazepine, lamotrigine, felbamate) or inhibit both transient and persistent sodium currents (phenytoin, topiramate, valproate), but the magnitude of the latter effect is much weaker than that found for cenobamate [6].

## 4. Pharmacokinetics

After oral administration, cenobamate undergoes a very efficient absorption from the intestines, reaching 88%. Steady-state plasma concentrations of cenobamate require about 2 weeks of once-daily drug administration, the area under the curve and peak plasma concentration being proportionally elevated depending upon the dose of cenobamate within its therapeutic range of 100–400 mg daily. Between 1 and 4 h is median time to drug peak concentration, the volume of distribution being 40–50 L, following oral administration. Moreover, cenobamate binds to plasma proteins by 60% [17].

The drug is metabolized by oxidation via cytochrome P450 (CYP) enzymes, and mainly CYP2A6, CYP2E1, and CYP2B are involved, with other CYP types playing a minor role in this respect. Glucuronidation is another metabolic pathway of cenobamate via uridine 5′-diphospho-glucuronosyltransferase. Cenobamate was documented to inhibit CYP2C19 and, on the other hand, was a stimulator of CYP3A4 and CYP2B6 [11], possessing thus a potential for interaction with concomitant drugs. Indeed, some ASMs (lamotrigine, carbamazepine, clobazam, phenobarbital, and phenytoin) may need dose adjustments [16,18].

Cenobamate is excreted in urine in the form of metabolites (almost 89%) with only a small proportion of unchanged drug (less than 7%). Importantly, moderate hepatic or renal impairment may augment exposure to cenobamate by 2.3- or 1.5-fold, respectively [17].

## 5. Cenobamate in Animal Models of Seizures and Behavioral Tests

Cenobamate displayed anticonvulsant effects in a variety of seizure tests in rodents [6] (Table 1). The drug was also active in models of anxiety and neuropathic pain [16,19].

Melnick et al. [20] evaluated the effects of cenobamate (YKP3089; R-enantiomer), YKP3090 (S-enantiomer), and YKP1983 (racemate) in chemically induced {pentylenetetrazol (PTZ)—generalized or repeated clonic seizures; bicuculline—clonic/tonic (generalized) seizures; picrotoxin—clonic (generalized) seizures)} and electrically induced (maximal electroshock seizure (MES)—model of human generalized tonic–clonic seizures; 6 Hz psychomotor seizure test—drug-resistant psychomotor (focal) seizure model; hippocampal kindled rat model of focal epilepsy) models of seizures. In addition, the effect of the drug on spike-and-wave seizures was studied in an animal model of absence seizures (Genetic Absence Epilepsy Rat from Strasbourg (GAERS). A rotarod test was used to assess the drug’s toxicity and its effect on motor coordination. According to the authors, “Minimal motor impairment (MMI; NINDS study) was assessed in rats by visual observation of gait, stance, placing response, exploratory behavior, and muscle tone”.

All substances tested (ip or po) mostly showed efficacy in a dose-dependent manner in controlling seizures and preventing seizure onset and spread. No protection was observed for cenobamate in the bicuculline (ip) model. In contrast, other compounds YKP3090 and YKP1983 showed efficacy in mouse and rat generalized seizure models (MES, PTZ), but no activity was observed in the rat hippocampal kindling model. In the rat GAERS model, ip-administered cenobamate at doses of 20 or 30 mg/kg exerted significant dose-dependent reductions in the number and cumulative duration of spike-and-wave discharges (SWDs), with a near-maximal effect observed at 30 mg/kg [20,21]. Notably, cenobamate increased the seizure threshold in the PTZ model. In addition, this ASM inhibited limbic seizures in a psychomotor convulsion model (6 Hz) at all three stimulus intensities evaluated [20,21].

In the rotarod test, the TD50 (a 50% neurotoxic dose) was determined for cenobamate and its enantiomers—YKP3090 and YKP1983. The former yielded a TD50 value in the range of 52 to 58 mg/kg after ip administration, while a TD50 of 85.6 mg/kg was obtained when the drug was administered via the po route; the times of peak activity in mice were 0.5 and 2 h, respectively, after cenobamate administration. In comparison, the median ip TD50 for YKP1983 was 92.5 mg/kg, and the time of its peak activity in mice was 15 min. Further, the median ip TD50 for YKP3090 was 143 mg/kg, and its peak activity in mice was also observed within 15 min [20].

Song et al. [22] evaluated the effects of cenobamate on cognitive behaviors and hippocampal long-term potentiation (a pattern of synaptic activity) in C57BL/6N mice. In the first stage, the drug was administered at a dose of 30 mg/kg (ip), which resulted in a reduction in locomotor activity, as assessed by an open field test (OFT). At 30 mg/kg, cenobamate exerted anxiolytic effects in the elevated zero-maze (EZM) test and did not affect despair-like behaviors in the tail suspension test. Also, this ASM did not modify social behaviors, as evaluated in the three-chamber social behavior test. Effects of cenobamate (30 mg/kg) on learning and memory were evaluated in novel object recognition (NOR) and object location memory (OLM) tests. In the NOR test, mice pretreated 24 h with cenobamate were shown to possess impaired discrimination between novel and familiar objects when compared to the acquisition phase carried out a day before. Similarly, cenobamate-pretreated mice exhibited a reduced ability to discriminate between moved and unmoved objects in the OLM test. At a lower dose of 10 mg/kg, cenobamate did not influence locomotor activity in the OFT but still exerted anxiolytic effects and impaired discrimination. When given chronically at 5 mg/kg, cenobamate exerted significant effects in EZM and NOR tests only. When administered chronically at 5 mg/kg, the drug was also documented to reduce the performance of mice in the Morris water maze test for evaluating spatial learning and memory [22].

Witherspoon et al. [23], considering that a number of ASMs (clonazepam, phenobarbital, phenytoin, valproate or vigabatrin) produced apoptopic neurodegeneration in the developing rat brain at clinically relevant concentrations [24], evaluated the antiseizure and neurotoxic effects of cenobamate (14–56 mg/kg) in neonatal rats. The results clearly indicate that cenobamate provided a clear-cut protection against pentylenetetrazol-induced convulsions and, moreover, did not display any neurotoxic potential similar to phenobarbital (75 mg/kg), which was responsible for neuronal death in lateral thalamus, hippocampus, septum, motor and cingulate cortices [23].

In conclusion, on the basis of experiments conducted on a number of animal models, the authors unequivocally point to the highly beneficial and effective profile of cenobamate [20,22,23]. The aforementioned mechanism of action of the drug clearly indicates a reduction in neuronal excitability, resulting in an antiseizure effect [22]. In addition, the results of experiments conducted by Witherspoon et al. [23] indicate that cenobamate may prove both effective in the treatment of epileptic seizures in neonates and safe for the youngest group of patients.

The above data clearly justify the need for further studies at the clinical level in patients with both generalized seizures and absence seizures in different age groups [20,22,23].

**Table 1:** Anticonvulsant effects of cenobamate and its enantiomers in various seizure tests.

Substance/Drug	Convulsion Model		Animal Model	Method of Administration	ED50 (mg/kg)	Anticonvulsant Effect	Bibliography
Cenobamate	PTZ (85 mg/kg)		mice	ip	28.5	+	[19,20]
			mice	po	7.1	+	[20]
	PTZ		rats	ip	13.6	+	
			rats	po	25	+/−	
	MES		mice	ip	9.8	+	[19,20]
			mice	po	3.3	+	[20]
			rats	ip	2.9	+	
			rats	po	1.9	+	
	6 Hz	22 mA	mice	ip	11	+	[19,20]
		32 mA			17.9	+	
		44 mA			16.5	+	
	picrotoxin		mice	ip	34.5	+	[20]
	bicuculline		mice	ip	>70	−	[20]
	hippocampal-kindling-induced seizures		rats	ip	16.4	+	
	audiogenic seizures		DBA/2 mouse model	ip	*	−	[25]
YKP3090	PTZ (85 mg/kg)		mice	ip	56.4	+	[20]
	PTZ		rats	ip	16.7	+	
	PTZ		rats	po	8.14	+	
	MES		mice	ip	38.2	+	
	MES		rats	ip	26.1	+	
	MES		rats	po	11.9	+	
	hippocampal-kindling-induced seizures		rats	ip	>50	−	[20]
YKP1983	PTZ (85 mg/kd)		mice	ip	35.9	+	
	PTZ		rats	ip	19.3	+	[20]
	PTZ		rats	po	12.3	+/−	
	MES		mice	ip	15.8	+	
	MES		rats	ip	8.4	+	
	MES		rats	po	2.9	+	
	hippocampal-kindling-induced seizures		rats	ip	30	−	

+, effective; +/−, only partially effective; −, not effective; MES, maximal electroshock model; PTZ, pentylenetetrazol model; *, cenobamate given ip at 5 mg/kg; ip, intraperitoneally; po, orally.

## 6. Cellular Effects of Cenobamate

From a cellular perspective, Song et al. [22] revealed that this ASM “enhanced inhibitory postsynaptic potentials by prolonging inhibitory postsynaptic current (IPSC) decay without affecting presynaptic GABA release or the peak amplitude of IPSCs”. On the other hand, experiments documented that cenobamate “suppressed hippocampal excitatory synaptic transmission by reducing the excitability of Schaffer collaterals and interfered with the induction of long-term potentiation”. Such a reduction in neuronal excitability was associated with an increase in action potential (AP) threshold, “which progressively increased in later APs during repetitive firing, indicating the activity-dependent modulation of neuronal sodium currents”. The authors noted that the drug suppressed neuronal cell excitability where GABA-ergic neurotransmission had an excitatory effect, and the cenobamate used rapidly increased the phosphorylation of eukaryotic elongation factor 2 in both the hippocampus of adult and neonatal mice [22].

## 7. Clinical Efficacy

One of the first multicenter clinical trials was conducted by Krauss et al. [26]. The study included 107 epilepsy and neurology centers in 16 countries. The patients (aged from 18 to 70 years) suffering from focal seizures, despite taking 1–3 ASMs, were randomized into four groups receiving either add-on cenobamate at daily doses of 100 (N = 108), 200 (N = 110), and 400 mg (N = 111) or placebo (N = 108) after a baseline evaluation for 8 weeks. A 12-week maintenance phase was preceded by 6-week titration. Changes in 28-day focal seizure frequency from baseline were assessed as a measure of the drug’s efficacy. Median percentage reductions in seizure frequency in all groups were observed—35.5% for the group receiving 100 mg daily, 55% for 200 mg, 55% for 400 mg, and 24% for the placebo group. Responder rates were 40, 56, 64, and 25%, respectively. As for adverse events, they were evident in 65, 76, 90, and 70% of the patients, respectively. Respective discontinuation of the treatment due to the adverse reactions occurred in 10, 14, 20, and 5% of the patients. One serious case (eosinophilia with systemic symptoms) was noted only in the group receiving cenobamate at 200 mg/kg [26].

In the second randomized study, phase 2, adjunctive cenobamate was given at 200 mg daily to patients (18—65 years) with focal seizures resistant to 1–3 ASMs [27]. The patients were randomized to placebo (N = 109) and cenobamate groups (N = 113). The 12-week trial consisted of a 6-week titration period, followed by a 6-week maintenance phase. Percent change in seizure frequency was assumed as the primary outcome.

The cenobamate group showed a greater median reduction in seizure frequency, which was 55.6% vs. 21.5% in the placebo group. As for the responder rate, it was also higher (55.6%) in the cenobamate group compared to the placebo group (22.2%). Interestingly, 28.3% of patients receiving cenobamate were seizure-free, whilst only 8.8% of patients were seizure-free in the placebo group. Among adverse effects, somnolence was observed in 22.1% of patients in the cenobamate group, dizziness in 22.1%, headache in 12.4%, nausea in 11.5%, and fatigue in 10.6% vs. 11.9, 16.5, 12.8, 4.6, and 6.4%, respectively, in the placebo group [27].

Open-label extension of the former trial examined long-term retention rate (up to 7.8 years), safety, and tolerability of adjunctive cenobamate [28]. Notably, during this study, both cenobamate and other ASMs could be adjusted to optimal doses, and a total of 149 patients participated: 85 for 6.25 years, and 64 for 6.8 (ranging from 6.4 to 7.8 years). The median dose of cenobamate was 200 mg daily with a range of 50–400 mg. The most encountered reasons for treatment discontinuation were patient withdrawal (19.5%), adverse effects (10.1%), and lack of efficacy (5.4%). The most frequent adverse events were dizziness (32.9%), headache (26.8%), and somnolence (21.5%), with their severity usually being mild or moderate [28].

A large, phase 3, multicenter, open-label safety study was carried out by Sperling et al. [29] in patients aged 18–70 years with focal seizures, taking 1–3 ASMs. The initial dose of adjuvant cenobamate was 12.5 mg daily, and it was elevated at 2-week intervals to 25, 50, 100, 150, and 200 mg daily. In some patients, a dose of 400 mg daily was administered. Also, in some patients, during the cenobamate titration period, doses of phenytoin or phenobarbital were reduced by 25–33%. Out of 1347 patients enrolled, after median treatment duration of 9 months, 269 patients discontinued their therapy mainly because of adverse events (137 patients) and other reasons (74 patients). Most frequent adverse effects were somnolence (observed in 28.1% of patients), dizziness (23.6%), and fatigue (16.6%). In 108 patients (8.1%), serious adverse events were noted: seizure (14 patients), epilepsy (5), pneumonia (4), fall (4), and dizziness (4). In no case was DRESS observed [29].

A multicenter and retrospective study was carried out in 14 Spanish hospitals on 170 patients with focal seizures, aged at least 18 years [30]. The endpoints assumed at least 50, 75, 90 or 100% seizure reductions as well as increases in seizure frequency after 3, 6, and 12 months, and at the last visit following an add-on therapy with cenobamate. Median epilepsy duration at baseline was 26 years, and median seizure number per month reached 11.3. The median number of concomitant ASMs was 3, and the most frequent were lacosamide, brivaracetam, carbamazepine, and clobazam. Cenobamate was administered in daily mean doses of 176, 200, and 250 mg at 3, 6, and 12 months. The proportion of seizure-free patients at the last visit was 13.3%, with responder rates of at least 90, 75, and 50% being 27.9, 45.5, and 63%, respectively. Notably, in 44.7% of patients, the number of concomitant ASMs was decreased. At 3 months, the cumulative percentage of patients with adverse effects were 68.2%, 74.1% at 6 months, and 74.1% at 12 months. Adverse effects leading to cenobamate discontinuation were observed in 3.5, 4.1, and 4.1% patients, respectively. Somnolence and dizziness were the most frequently noted adverse effects [30].

Lattanzi et al. [1] provided a network meta-analysis on the efficacy and safety of a number of ASMs (brivaracetam, cenobamate, eslicarbazepine acetate, lacosamide, and perampanel) in patients with focal-onset seizures. The meta-analysis was based upon sixteen trials enrolling 4507 patients who were randomized to adjunctive ASMs as mentioned above. Cenobamate proved to be the most efficient ASM, reducing seizure frequency by at least 50%. However, this relationship was no longer seen when seizure freedom was considered. Cenobamate seems thus the best option for the reduction in seizure frequency, whilst brivaracetam and lacosamide appear to be the best-tolerated ASMs. According to Sander et al. [31], cenobamate exhibited high retention rates in the open-label studies, reaching 72% at 2 years.

In their review on recently approved ASMs (cenobamate for focal epilepsy and fenfluramine for Dravet syndrome), Klein et al. [32] pointed out some important facts. First of all, they underlined encouraging results from clinical trials showing the distinct efficacy of this ASM based on data derived from clinical trials. Adjunctive cenobamate was able to achieve 21% seizure freedom and reduce seizure frequency in a significant proportion of patients with focal epilepsy, as shown in a randomized trial. Moreover, the proportion of seizure-free patients for about 30–45 months ranged from 10 to 36% in long-term, open-label trials. The adverse effects of cenobamate were comparable to those induced by other ASMs. Thus, the authors were of the opinion that cenobamate may offer the most effective therapy of focal seizures, but a crucial question arises as to why this ASM is not as frequently used as it should be. Some reasons may be limited knowledge about completely novel drugs, which may be linked to inadequate post-launch information [32].

Villanueva et al. [33] decided to compare the clinical efficacy of a number of third-generation ASMs, including cenobamate, with the use of the Number Needed to Treat (NNT) in patients with drug-resistant focal seizures. NNT data were calculated from the ≥50% and 100% responder rates taken from clinical trials with cenobamate, brivaracetam, perampanel, lacosamide, and eslicarbazepine acetate. The results clearly indicated that cenobamate had the lowest NNT value for ≥50% and 100% responder rates when compared with other ASMs. Moreover, this ASM (at ≥50% responder rate) possessed the lowest cost for NNT at daily defined dose, and lacosamide and eslicarbazepine acetate at minimum and maximum doses, respectively. When a 100% responder rate was considered, cenobamate proved to possess the lowest cost for NNT at daily defined and the maximum dose, lacosamide being the most profitable ASM at the minimum dose [33].

Recent clinical studies on cenobamate highlight its promising efficacy and tolerability. In patients with focal-onset seizures, failing to respond satisfactorily to at least two ASMs (lacosamide, perampanel or brivaracetam), add-on cenobamate resulted in seizure freedom in 14% of patients (N = 150) after one year, the 50% responder rate being 61% [34]. In another study involving 40 patients with uncontrolled focal epilepsy, adjunctive cenobamate improved seizure control, and the number of concomitant ASMs could be reduced from three to two after 12 weeks [35].

Initial studies seem to indicate that the antiseizure potential of cenobamate may also be extended to other seizure types. Agashe et al. [36] evaluated the efficacy of cenobamate in two groups of patients: (1) with genetic generalized epilepsies (N = 4); and (2) in patients with a combination of generalized and focal epilepsies, including Lennox–Gastaut syndrome and Dravet syndrome (n = 9). In the first group, cenobamate provided a >50% reduction in seizure activity. In the second group, the occurrence of both seizure types was reduced. Finally, in both groups, a mean reduction of 58%, regarding all types of seizures, was observed, with ≥50% responder rate of 70%. No worsening of generalized-onset seizures occurred in either cohort. Seventy-seven percent of patients experienced side effects, warranting a modification of treatment managed by slower titration, dose reduction of cenobamate, or discontinuation of other ASMs [36]. The clinical efficacy of cenobamate is displayed in Table 2.

In conclusion, based on the results obtained in clinical trials, it has been clearly demonstrated that cenobamate as an adjunctive drug is safe and well tolerated by patients, including in long-term studies [28,29]. The studies conducted included adult patients with seizure types such as uncontrolled focal seizures [26,27], drug-resistant focal seizures [31,33,34,35], generalized tonic–clonic seizures and combined generalized and focal epilepsy [36]. In all cases, a significant reduction in the number and frequency of epileptic seizures was observed. No increase in seizure activity was observed in any group. Side effects were of generally mild to moderate severity and were associated with the higher doses used. Among them, the most frequently mentioned were drowsiness, dizziness and headache, and nausea [26,27,28,29,30,31,32,33,34,35,36]. In a small number of cases, side effects led to treatment discontinuation [28,29,30,31]. The authors unanimously concluded that cenobamate is an ASM with great therapeutic potential, safe and well tolerated by patients, while longer, slower dosage titration and possibly a reduction in the drug dose allow for limiting of potential side effects.

## 8. Cenobamate—Liver and Kidney Functioning

Currently, there are few publications on the effects of cenobamate treatment on organs such as the liver or kidneys, and the available results mostly indicate a fairly safe profile for this ASM. To date, it has been observed that cenobamate therapy is associated with a low risk (in 1–4% of patients) of elevation in serum liver enzymes. These changes are mild to moderate and usually transient in nature [37]. No cases of clinically overt liver damage with jaundice have been reported in pre-market clinical studies of cenobamate, but on the other hand, there are known cases of liver damage, usually in the form of multi-organ hypersensitivity syndrome, such as drug reaction with eosinophilia and systemic symptoms (DRESS) [38,39]. Such a condition was observed in 3 of 953 patients, which occurred within the first 3 to 6 weeks. It is noteworthy that, in these patients, the initial titration was quite rapid—4 to 6 weeks [39]. In comparison, another large (more than 1000 patients) open study with a longer titration period (12 weeks) reported no cases of DRESS syndrome [29]. The authors of the above papers unanimously concluded that a slower and longer drug titration period significantly reduces the risk of treatment side effects and increases drug tolerability [26,27,29,40].

Cenobamate is extensively metabolized in the liver by cytochrome 450 enzymes and undergoes processes such as glucuronidation, hydrolysis, and hydroxylation. The drug is a weak inhibitor of CYP 2C19 and an inducer of CYP 2B6, 2C8, and 3A4, which, in practice, means that it can induce drug–drug interactions [41].

Vernillet et al. [41] evaluated the absorption, metabolism, and excretion of cenobamate following a single 400 mg dose containing 50 μCi of [14C]-cenobamate. The study was conducted in healthy men, who were administered the drug in capsule form by the oral route. The results showed that this ASM was rapidly and extensively absorbed (median time to maximum plasma concentration of 1.25 h) and limited penetration of both the drug itself and its metabolites into red blood cells (RBCs). At this point, it was possible to identify the following metabolites: cenobamate and N-glucuronide (M1) in plasma; in turn, urine analysis showed the presence of eight metabolites: M1, O-glucuronide (M2a), O-glucuronide (M2b) regioisomer, side chain O-glucuronide (M3), glucuronide of dihydrodiol (M5), dihydrodiol (M6), dihydrodiol diastereomer (M7), and N-glucuronide-tetrazole (M11). Two trace metabolites in urine, designated P2 and P5, could not be identified. Six metabolites were found in feces, including minor (less than 0.5%) cenobamate, and ≤1.75% each of M1, M3, M6, M7, and M11. In addition, the authors found that all metabolites were formed slowly, and those recovered from the urine accounted for 88% of the dose used, suggesting that the drug was mainly metabolized by the kidneys, while metabolites from the plasma and liver were eliminated very quickly, and cenobamate appeared to be the main component circulating in the plasma after oral administration. Further studies, including multiple doses, showed that cenobamate is generally safe and well tolerated after single doses of up to 750 mg and when used in multiple doses in the range of 50–500 mg/day [41].

In summary, no side effects or deaths were observed in patients during the study. Only one patient had elevated creatinine kinase values, which returned to normal by the end of the study. Other hematological as well as renal parameters remained normal in all patients. This allowed us to conclude that cenobamate is safe and well tolerated. [37,41]. For patients burdened with additional renal and/or hepatic impairment (especially patients with mild to moderate and severe renal impairment and patients with mild to moderate hepatic impairment), the authors suggest caution and reducing cenobamate doses. For patients with end-stage renal failure and severe hepatic impairment, there are no data available on the drug’s pharmacokinetics, doses used or possible adverse effects of therapy.

## 9. Drug Load, Overtreatment of Epilepsy, and Drug Interactions

Generally, therapeutic drug monitoring is assumed to be routinely carried out in patients receiving classical ASMs [42]. However, this procedure may be also recommended with newer ASMs (for instance, lamotrigine or levetiracetam) for pediatric patients or patients prescribed polytherapy [43]. The total drug load may be quantified basing on the defined daily dose which reflects average maintenance dose per 24 h. The drug load for a single ASM may be thus calculated as the ratio of actual daily dose to defined daily dose. The total drug load is the sum of single drug loads in an individual patient [44]. Certainly, the drug load in patients on polytherapy may be quite significant, especially when four or five ASMs have to be taken. Hilgers and Schaefer [45] analyzed adverse effects of newer ASMs in 562 patients with mainly focal (79.4%) and generalized seizures (14.8%). Only 10.1% of patients received monotherapy, 34.2% were on duotherapy, 36.5% were prescribed three, 15.7% prescribed four, 3.4% prescribed five, and only 0.2% were prescribed six ASMs. Interestingly, the total mean drug load was 1.15 for patients on monotherapy, 2.42 for duotherapy, 3.33 for three ASMs, 4.50 for four, and 5.56 for patients prescribed five ASMs [45]. According to Deckers [46], overtreatment is quite probable in polytherapy and may be associated with more adverse events. Indeed, Hilger and Schaefer [45] have found that the occurrence of adverse effects was correlated with the total drug load and levetiracetam, compared to lacosamide, oxcarbazepine, pregabalin, topiramate, and zonisamide, exhibited the best tolerance. Rational polytherapy assumes optimizing anticonvulsant efficacy and minimizing adverse events [47]. The anticonvulsant efficacy of ASMs is obviously dependent on whether combinations of ASMs exert synergistic, additive, or antagonistic interactions. Experimental and clinical evidences clearly indicate that combining ASMs with different mechanisms of action is associated with anticonvulsant synergy with frequent neurotoxic antagonism, while such benefits do not accompany combinations of ASMs with single comparable mechanisms of actions [48,49]. Preclinical data point to lamotrigine + valproate, lamotrigine + topiramate, tiagabine + oxcarbazepine as beneficial combinations (anticonvulsant synergy and neurotoxic antagonism) and, in contrast, to lamotrigine + oxcarbazepine as an example of an irrational combination [48,49]. The clinical data are scarce and favor lamotrigine + valproate (result from controlled studies) as a very effective combination [50], supporting the view on the beneficious combinations of ASMs with different mechanisms of action. The first study on the interaction of cenobamate with other ASMs in audiogenic seizures in mice indicates that the anticonvulsant efficacy of diazepam, clobazam, levetiracetam, perampanel, phenobarbital, topiramate, and valproate was potentiated [25]. In addition, these interactions were pharmacodynamic as cenobamate did not affect the brain concentrations of these ASMs. However, the anticonvulsant activity of sodium channel inhibitors (carbamazepine, oxcarbazepine, and phenytoin) was not affected by cenobamate [25].

In a real-world setting, cenobamate was given to 54 patients with focal-onset drug-resistant epilepsy, out of whom 49 completed the study [51]. At the start, 24 patients were prescribed carbamazepine, 22 clobazam, and 17 lacosamide. In 17 patients, cenobamate was administered at a low dose (<200 mg daily). At the last follow-up visit, median seizure reduction reached 69.5%. Specifically, in 32 patients (59.2%), a ≥50% seizure reduction and, in 20 patients (42%), a reduction of ≥75% was achieved, with 10 patients (20.2%) being seizure-free [51]. Another study with the same approach revealed that cenobamate at a median dose of 200 mg daily diminished the intensity of highly refractory focal seizures in 54.3% of subjects (N = 51 with a retention rate of 80.4% at the last follow-up). On inclusion of cenobamate, the patients were receiving sodium channel blockers (54.9%) and ASMs enhancing GABA-ergic transmission (37.3%). Interestingly, the number of sodium channel blockers could be reduced in the presence of cenobamate [52]. A real-world retrospective study by Peña-Ceballos et al. [49] analyzed cenobamate (median dose of 250 mg, range 75–350 mg daily) as an add-on ASM in 57 patients with ultra-refractory focal epilepsy. Most common concomitant ASMs at baseline were clobazam (in 59.6% of subjects), eslicarbazepine (27%), lamotrigine (25%), and brivaracetam (16%). The results indicated that 5.3% of the patients became seizure-free, 42.1% achieved a 75–99% reduction in seizure activity, and 28.1% experienced a reduction of 50–74% in seizures. Remarkably, among responding patients, 67.4% of them received at least 250 mg of cenobamate per day [53]. In 95 patients with mainly focal epilepsy (86.3%), cenobamate was given (at different doses of 25–100 mg daily) as an add-on therapy for at least 3 months at data cutoff [54]. In five patients, cenobamate was withdrawn mainly because of adverse effects. At data cut-off, 50% of patients could discontinue at least one ASM, usually clobazam, levetiracetam, and phenytoin. In some patients (23.3%), doses of concomitant ASMs (mainly phenytoin and clobazam) could be reduced, and interestingly, 16 patients could be on cenobamate monotherapy. Of the 79 patients who received cenobamate for more than 3 months at data cut-off, 51.9% were seizure-free. The most common adverse effect observed in 7 patients was fatigue [54]. Further data on a possibility to reduce concomitant ASM load are available. Aboutamar et al. [55] evaluated 240 patients with uncontrolled focal seizures in an open-label study; cenobamate was added to the existing ASMs (from 1 to 3) in an initial dose of 25 mg, the target dose being 200 mg daily. Within one year, the mean drug load of concomitant ASMs was reduced by 29.4%, and after 2 years by 31.8%. The most significant reduction (55.2%) was observed in benzodiazepines, but dosages of ASMs representing other mechanisms of action could also be decreased. Importantly, the reduced concomitant ASM drug load was not associated with worse response rates [55]. There is a Spanish consensus aimed at a reduction of doses of concomitant ASMs in patients with drug-resistant focal epilepsy scheduled for cenobamate as an add-on drug [56]. The expert panel is of the opinion that in patients treated with high doses of sodium channel blockers (for instance, carbamazepine at ≥800 mg or lamotrigine at ≥300 mg daily), the drug load may be reduced once cenobamate is given at ≥150 mg per day. Dose adjustments may be started earlier in case of severe adverse effects. With respect to ASMs enhancing GABA-mediated events, especially clobazam must be taken into consideration.

## 10. Conclusions

Cenobamate exhibits a unique mechanism of action when its particular effects on persistent sodium current and extrasynaptic GABA-ergic events are taken into consideration. Possibly, this ASM may also share a yet-undiscovered mechanism.

Cenobamate is one of the newest ASMs, receiving enormous interest, reflected in a considerable number of original and review articles, also including meta-analyses. The drug has been licensed in the form of an add-on therapy for focal refractory epilepsy. Cenobamate has proved to be associated with a high response rate and seizure freedom, usually higher when compared to other newer ASMs. Many review papers, also not mentioned above, underline its significant efficacy with a low proportion of patients discontinuing the antiseizure therapy due to adverse events [12,16,57,58,59,60]. It is also of importance that upon addition of cenobamate to the existing ASMs, most of patients exhibited stable or even better cognitive performance [61]. Neuroprotective effects of cenobamate must also be considered as a possible mechanism for the epilepsy therapy [62].

Although licensed for focal epilepsies, cenobamate also possesses a potential for other seizure types as indicated by an initial study [36]. Further studies are needed to confirm this result. Nonetheless, as suggested by French [63] and Schmitz et al. [12], cenobamate may be considered a game changer in the therapy of drug-resistant focal epilepsy.

## 11. Future Prospects

Preclinical studies as a basis for clinical trials and introduction of rational monotherapy/polytherapy (including combination with cenobamate) in patients with epilepsy should be considered:

(1) Experimental studies, especially isobolographic analysis, allow for clearly identifying drug interactions resulting in synergy, additivity, or antagonism of action. In practice, this makes it possible to choose the best option for polytherapy (especially in the case of using ASMs with different mechanisms of action), characterized by synergy or additivity in terms of therapeutic effects and antagonism in the case of adverse effects of the drugs combined [48].
(2) Experimental studies provide an opportunity to learn more about the efficacy of cenobamate in patients with types of seizures other than focal [36], which will help expand the clinical recommendation for this ASM.
(3) Preclinical studies indicate the neuroprotective potential of cenobamate, which, in practice, may indicate an ASM limiting the process of epileptogenesis and, as a result, positively modifying the course of epilepsy [62].
(4) Preclinical studies have shown the lack of neurotoxicity caused by cenobamate in the brains of newborns [23], making it possible to use this AMS safely in neonatal seizures.

## Figures and Tables

**Table 2:** Examples of clinical efficacy of cenobamate in the form of an add-on therapy.

Clinical Trial	Age of Patients [Years]	Type of Epileptic Seizures	Dosage of Cenobamate [mg/24 h]	Duration of Therapy	Median Percentage Reductions in Seizure Frequency [%]	Responder Rates [%]	Frequency of Adverse Events [% of Patients]	Respective Discontinuation of the Treatment [% of Patients]	References
NCT01866111	18–70	resistant focal seizures	placebo	8 weeks	24	25	70	5	[26]
			100		35.5	40	65	10	
			200		55	56	76	14	
			400		55	64	90	20	
NCT01397968 phase 2	18–65	resistant focal seizures	placebo	12 weeks	21.5	21.5	≤16.5	NA	[27]
			200		55.6	55.6	≤22.1	NA	
Open-label extension of the trial (NCT01397968) examined long-term retention rate	18–65	resistant focal seizures	200 mg daily with a range of 50–400 mg	6.4–7.8 years	NA	NA	≤32.9	10.1–19.5	[28]
YKP3089C021, phase 3	18–70	resistant focal seizures	25–400	NA	NA	NA	≤28.1	<20	[29]

NA, no data available.

## Data Availability

The data presented in this study are available on request from the corresponding author.
